# Assessing cancer patients’ quality of life and supportive care needs: Translation-revalidation of the CARES in Flemish and exhaustive evaluation of concurrent validity

**DOI:** 10.1186/s12913-016-1335-4

**Published:** 2016-03-11

**Authors:** Bojoura Schouten, Elke Van Hoof, Patrick Vankrunkelsven, Ward Schrooten, Paul Bulens, Frank Buntinx, Jeroen Mebis, Dominique Vandijck, Irina Cleemput, Johan Hellings

**Affiliations:** Faculty of Medicine and Life Sciences, Hasselt University, Martelarenlaan 42, 3500 Hasselt, Belgium; Department of Experimental and Applied Psychology, Faculty of Psychological and Educational Sciences, Free University of Brussels, Pleinlaan 2, 1050 Elsene, Belgium; Department of Public Health and Primary Care, Faculty of Medicine, KU Leuven, Kapucijnenvoer 33, PB 7001, 3000 Leuven, Belgium; Belgian Center for Evidence-Based Medicine (CEBAM), Kapucijnenvoer 33- blok J, 3000 Leuven, Belgium; Jessaziekenhuis, Stadsomvaart 11, 3500 Hasselt, Belgium; ICURO, Guimardstraat 1, 1040 Brussel, Belgium; KCE - Belgian Health Care Knowledge Centre, Kruidtuinlaan 55, 1000 Brussel, Belgium; AZ Delta, Rode-Kruisstraat 20, 8800 Roeselare, Belgium

**Keywords:** Cancer, Psychosocial, Quality of life, Distress, Needs assessment, Validation, Supportive care

## Abstract

**Background:**

The prevalence of cancer increases every year, leading to a growing population of patients and survivors in need for care. To achieve good quality care, a patient-centered approach is essential. Correct and timely detection of needs throughout the different stages of the care trajectory is crucial and can be supported by the use of screening and assessment in a stepped-care approach. The Cancer Rehabilitation Evaluation System (CARES) is a valuable and comprehensive quality of life and needs assessment instrument. For use in Flemish research and clinical practice, the CARES tool was translated for the Dutch-speaking part of Belgium (Flanders) from its original English format. This protocol paper describes the translation and revalidation of this Flemish CARES version.

**Methods:**

After forward-backward translation of the CARES into Flemish we aim to recruit 150 adult cancer patients with a primary cancer diagnosis (stage I, II or III) for validation. In this study with a combination of qualitative and a quantitative approach, qualitative data will be collected through focus groups and supplemented by two phases of quantitative data collection: i) an initial patient survey containing questions on socio-demographic and medical data, the CARES and seven concurrent instruments; and ii) a second survey administered after 1 week containing the CARES and supplementary questions to explore their impressions on the content and the feasibility of the CARES.

**Discussion:**

With this extensive data collection process, psychometric validity of the Flemish CARES can be tested thoroughly using classical test theory. Internal consistency of summary scales, test-retest reliability, content validity, construct validity, concurrent validity and feasibility of the instrument will be examined. If the Flemish CARES version is found reliable, valid and feasible, it will be used in future research and clinical practice. Comprehensive assessment with the CARES in a stepped-care approach can facilitate timely identification of cancer patients’ psychosocial concerns and care needs so it can contribute to efficient provision of patient-centered quality care.

**Trial registration:**

ClinicalTrials.gov: NCT02282696 (July 16, 2014).

**Electronic supplementary material:**

The online version of this article (doi:10.1186/s12913-016-1335-4) contains supplementary material, which is available to authorized users.

## Background

The diagnosis of cancer has an enormous impact on people’s lives. In additional to the threat on one’s physical health, cancer patients are confronted with psychosocial problems and care needs [1–13]. Timely and accurate detection of those psychosocial problems and care needs is of great importance to offer more patient-centered care, efficient referral and to prevent comorbid psychopathology [14–18].Simple quality of life (QOL) measurement and distress-screening are popular methods to explore people’s psychosocial well-being [9, 19–22]. In contrast, needs assessment is a strategy that focuses on identifying the unresolved concerns that patients are experiencing and determines if they desire further assistance throughout the continuum of care [23]. Indeed, not all patients experiencing distress or reduced QOL and need professional support from the care system [24]. Needs assessment can provide important input from the patients’ perspective and guide appropriate intervention in the multidisciplinary process of care. As a result of patient-report data, health care resources can be allocated in the most appropriate way. The use of needs assessment can therefore contribute to patient-centered quality cancer care [14, 25–27].

To our knowledge there are no validated Flemish needs assessment tools available. Therefore, this study will be dedicated to the validation of a needs assessment tool for use in Belgian research and clinical practice. To provide a good understanding of psychosocial healthcare needs, the content of a needs assessment tool should be comprehensive enough to benefit multidisciplinary stakeholders involved in cancer care i.e. medical specialists, nursing, psychologists, social welfare workers, general practitioners, health insurance agencies. In the search for an appropriate needs assessment tool, the following criteria were used: 1) the instrument should be generic across tumor type and staging, i.e. suitable in all cancer patients; 2) the assessment should encompass the bio-psychosocial impact of the disease and treatment on patients’ overall well-being i.e. physical, emotional, cognitive, social, relational, sexual and financial, their daily functioning and the potential resulting care needs; and, 3) the tool should have a proven psychometric robustness, demonstrating good reliability and validity, and be feasible for patients.

Several review studies describing needs assessment tools for adult cancer patients are available [28–30]. From the tools discussed 24 instruments are patient-reported outcomes (PRO) for adult patients with any type of cancer. These needs assessment tools and associated psychometric properties are presented in brief in Table 1 (and in full in Additional file 1).

**Table 1:** Summary of needs assessment tools and psychometric properties

Instrument	Validity		Reliability		Responsiveness	Feasibility		
	Content Validity	Other types of validity	Internal consistency	Reproducibility		Time, Reading Level, Acceptability		
CaNDI	+	+	+	+	-	T: -	RL: +	A: -
CARES	+	+	+	+	-	T: +	RL: -	A: +
CARES-SF	+	+	+	+	+	T: +	RL: -	A: -
CCM	+	+	+	+	-	T: +	RL: +	A: +
CHOICEs	+	-	+	-	-	T: +	RL: -	A: +
Concerns checklist	+	+	-	-	-	T: -	RL: -	A: -
CNAT	+	+	+	-	-	T: -	RL: -	A: +
CNQ-SF	+	+	+	-	-	T: +	RL: +	A: +
CPILS	+	+	+	-	-	T: -	RL: -	A: -
CPNS	+	-	+	-	-	T: +	RL: -	A: +
CPNQ	+	+	+	+	-	T: +	RL: +	A: -
Distress management tool	+	-	-	-	-	T: -	RL: -	A: -
INM	+	-	+	-	-	T: -	RL: -	A: -
NEQ	+	+	+	+	-	T: -	RL: -	A: +
OCPC	+	-	-	-	-	T: -	RL: -	A: +
PINQ	+	+	+	-	+	T: -	RL: -	A: +
PNAS	+	-	+	-	-	T: -	RL: -	A: -
PNAT	+	+	+	+	-	T: +	RL: -	A: -
PNI	+	+	+	-	-	T: -	RL: +	A: -
Problem checklist	+	+	+	-	-	T: -	RL: -	A: +
SCNS	+	-	+	-	-	T: +	RL: +	A: +
SCNS-SF34	+	+	+	-	-	T: -	RL: +	A: -
SNST	+	-	-	-	-	T: -	RL: +	A: +
Symptoms and concerns checklist	+	+	+	+	-	T: +	RL: +	A: +

+ : evidence for psychometric property

- : no evidence for psychometric property or evidence not available

*Abbreviations*: CaNDI (Cancer Needs Distress Inventory), CARES (Cancer Rehabilitation Evaluation System), CARES-SF (Cancer Rehabilitation Evaluation System-Short Form), CCM (Cancer care monitor), CHOICEs assessment (Creating better health outcomes by improving communication about patients’ experiences assessment), CNAT (Comprehensive needs assessment tool in cancer), CNQ-SF (Cancer Needs Questionnaire Short Form), CPILS (Cancer Problems in Living Scale), CPNS (Cancer Patient Need Survey) CPNQ (Cancer Patient Need Questionnaire), Distress management tool, INM (Information Needs Measure), NEQ (Need Evaluation Questionnaire), OCPC (Oncology Clinic Patient Checklist), PINQ (Patient Information Need Questionnaire), PNAS (Psychosocial needs assessment survey), PNAT (Patient Needs Assessment Tool), PNI (Psychosocial Needs Inventory), Problem checklist, SCNS (Supportive Care Needs Survey), SCNS-SF34 (Supportive Care Needs Survey Short Form), SNST (Supportive Care Needs screening Tool), Symptoms and concerns checklist

Among other tools, the Cancer Rehabilitation Evaluation System (CARES) was positively evaluated [28–30]. The CARES is a QOL and needs assessment instrument, developed to provide an efficient way of gathering specific information about the day-to-day problems and rehabilitation needs of cancer patients. The instrument can be used for research or clinical objectives and has been applied across cancer type and stage [31–44]. The 139 items of the CARES are placed under 31 subscales and represented according to six summary scales, as shown in Table 2. A copy of the original CARES questionnaire and patient score profile can be found in Additional files 2 and 3.

**Table 2:** CARES Summary scales and subscales

Summary scales (n items)	Subscales
Physical (26)	Ambulation Activities of daily living Recreational activities Weight loss Difficulty working Pain Clothing
Medical Interaction (11)	Problems obtaining info from medical team Difficulty communicating with medical team Control of medical team
Marital^a^ (18)	Communication with partner Affection with partner Interaction with partner Overprotection by partner Neglect of care by partner
Psychosocial (44)	Body image Psychological distress Cognitive problems Difficulty communicating with friends/relatives Friends/relatives difficulty interacting Anxiety in medical situations Worry Interaction with children^a^ At work concerns^a^
Sexual (8)	Sex interest Sexual dysfunction^a^
Miscellaneous (32)	Compliance Economic barriers Dating^a^ Chemotherapy-related problems^a^ Radiation-related problems^a^ Ostomy^a^ Prosthesis^a^ Miscellaneous items

^a^ Items may not apply to all patients

The psychometric robustness of the CARES and its earlier development versions (the Cancer Inventory of Problem Situations) are well documented [33, 34, 36, 45]. The results demonstrate that the CARES and its summary scales have excellent internal consistency (α = 0.87–0.94) and high test-retest correlations (*r* = 0.84–0.95). The instrument has moderate to high correlations with the Symptom Checklist-90 (SCL-90) [46], Dyadic Adjustment Scale (DAS) [47], Karnofsky Performance status Scale (KPS) [48, 49] and a visual analogue scale [50] for QOL before and after cancer, that were used to investigate concurrent validity. The content validity was supported with the results from post-administration interviews [35, 45].

Considering the CARES is reported as a valid and feasible tool that can be used for all cancer patients to assess a comprehensive range of bio-psychosocial aspects of well-being, this instrument was chosen to be translated and validated for further use in Flemish cancer care facilities and research.

The psychometric robustness of the Flemish CARES version will be tested thoroughly. We plan to evaluate the internal consistency of the CARES and its summary scales, the test-retest reliability will be considered, the construct validity will be explored, and the concurrent validity of the CARES and its summary scales will be checked with several comparative instruments. This paper describes the study protocol of this multi-stepped process.

## Methods

### Translation of the CARES

Belgium is a trilingual country with Dutch, French and German as official state languages. The Dutch language in Belgium, called Flemish, is slightly different from the Dutch language in the Netherlands in terms of vocabulary. Since current CARES translation is made for the Dutch-speaking part of Belgium, this paper refers to the Flemish CARES version only. We have no knowledge of a CARES translation appropriate for The Netherlands. However, there is a translation of the CARES-Short Form (CARES-SF) [51]. If one would like to use the full version of the CARES in The Netherlands, a revision of the translation would be needed.

The Flemish CARES version resulted from a forward-backward translation process with sworn translators and an expert group, following the guidelines for translating questionnaires described by Beaton et al. [52]. First, sworn translators translated the original US English CARES into Flemish. Two independent researchers revised the resulting texts for content fidelity and an expert group, comprised of professionals from the field of care management, oncology, primary care and psychology, agreed on the final Flemish version. The questionnaire was again translated back into English by sworn translators and the original CARES and English back-translation were compared by a native speaker, concluding the content of the questionnaire was maintained.

### Design of the study

A mix of qualitative and quantitative methods will be used for the validation study of the Flemish CARES.

Qualitative data collection, that will be used to evaluate *content validity* and *feasibility* of the instrument, will consist of conducting focus group discussions until data saturation is reached. We estimate that it will be necessary to arrange four or five focus group discussions with six to ten participants. The discussions will be facilitated with several key questions and transcribed afterwards for thematic content analysis. Further detailed description of this qualitative research activities will be part of another publication, since we prefer to focus on a detailed description of the quantitative research in this paper.

For the quantitative data collection, questionnaires containing the CARES and different complementary instruments (see further) will be used to evaluate *reliability, construct validity and concurrent validity.* This quantitative part of the validation study is described in further detail in this protocol.

### Sample size

There are no general criteria for the sample size in a validation study, but a sample size of at least 50–100 is generally recommended [53]. Sample sizes in the validation research of the original CARES varied for each psychometric quality (Table 3) [35]. Two large sample sizes of 479 and 1047 were used for the investigation of construct validity. Other aspects of reliability and validity were tested with sample sizes of 22 to 120 participants. Given the available time and resources, setting the goal to include 150 participants for this validation study of the Flemish CARES version is feasible. Considering the response rates of 40–60 % that are usually reached in the research domain of psycho-oncology, inviting at least 250 eligible patients is a conservative approach to guarantee the minimal amount of 150 participants.

**Table 3:** Sample sizes validation research original CARES

Psychometric quality	Analysis	Sample size (N)
Test-retest reliability	Correlations between CARES summary scores	71 120
	Rating agreement	71 120
Construct validity	Factor analysis on all items	479
	Second-order factor analysis on 31 subscales	479 1047
Concurrent validity	Correlation between CARES and SCL-90	87
	Correlation between CARES and SCL-90, DAS, KPS and QOL visual analogue scale	120
Sensitivity	CARES compared to clinical interview	22
	CARES compared to a needs assessment interview	24 64
Content validity Acceptability to patients	Questions on relevance of CARES content, completion time, understandability and acceptability items.	22 64

*Abbreviations CARES* Cancer Rehabilitation Evaluation System, *SCL-90* Symptom Checklist-90, *DAS* Dyadic Adjustment Scale, *KPS* Karnofsky Performance status Scale

### Study population and recruitment

The CARES was constructed to detect rehabilitation needs and QOL, with a secondary intent to stimulate patients’ competences and patient empowerment for increased involvement in their own rehabilitation. Therefore, only patients with a primary cancer diagnosis treated with a curative intent will be recruited for this validation study. Details on the in- and exclusion criteria are listed in Table 4.

**Table 4:** Inclusion and exclusion criteria for eligible patients

Inclusion criteria	Exclusion criteria
▪ Male and female cancer patients ▪ Primary stage I, II and III diagnosis ^a^ ▪ At different stages of the care process: recently diagnosed, currently undergoing treatment, and post-treatment in follow-up care. ▪ All types of cancer ▪ Aged between 25 and 60 years ^b^	▪ Having had or having premorbid neurological problems or cognitive dysfunctions ▪ The lack of proficiency in Flemish-Dutch

^a^ This criteria serves to exclude palliative patients, since we aim to include participants that have an perspective on rehabilitation

^b^ We believe the social context, role fulfillment, obligations and expectations differ between adolescents, adults and elderly resulting in other psychosocial concerns. To recruit adult cancer patients we chose the age range of 25–60 years

^c^ This makes a person unsuitable for participation in questionnaire research

Participants will be recruited from four Flemish hospitals (two public and two private, with a range from 340 to 1015 beds). In order to generate a representative research sample, several medical departments will conduct patient recruitment and include medical oncology, radiotherapy, gynecology, urology, and gastroenterology services.

### Study procedure

**Fig. 1:**
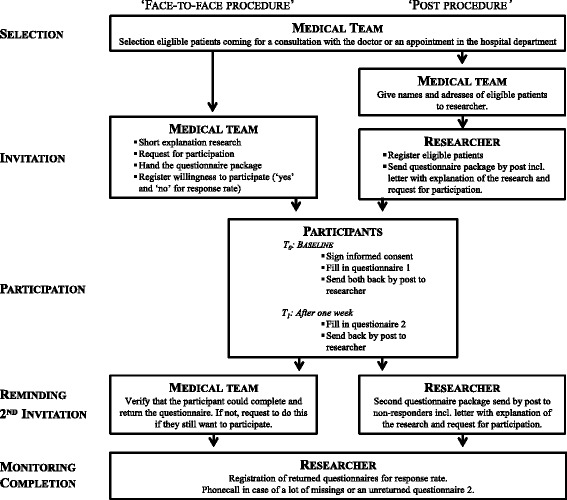
Study procedure

Eligible patients will be selected by the medical team according to the inclusion and exclusion criteria. Given the complexity of the clinical field and variable structures of the participating departments, two alternative procedures to invite patients to participate in the study will be used. On the basis of team organization and availability of time, the physician of the medical unit will choose to recruit patients with either the ‘face-to-face procedure’ or the ‘post procedure’. In the ‘face-to-face procedure’, a member of the medical team will explain the study briefly and invite the patient to participate. If the patient agrees to participate, he/she will immediately receive a study package with the informed consent form, a ‘what to do’-scheme, the first questionnaire and a stamped and addressed envelope to return the questionnaire. In the ‘post procedure’, eligible patients will be sent an identical study package by post, containing a short letter explaining the study, the informed consent form, a ‘what to do’-scheme, the first questionnaire and a stamped and addressed envelope to return the questionnaire. One week later participants have to fill in the second questionnaire, containing the CARES for test-retest reliability, and send it back in another stamped and addressed envelope provided. If the questionnaire is not sent back, the participants recruited via the ‘face-to-face procedures will be contacted by a team member and asked if they still want to participate and asked to return a completed questionnaire. Participants invited through post-procedure will be sent a reminder and second questionnaire package after 1 month. This study procedure is visualized in Fig. 1.

Participants will be contacted by phone or by e-mail when returned questionnaires have a large number of missing responses or if the second questionnaire is not received in the expected timespan. Ethical standards limit the number of participant contacts, there is a maximum of two attempts to contact a participant.

### Questionnaires

Data collected with the *first questionnaire* includes socio-demographic characteristics, medical characteristics, the CARES and several concurrent instruments measuring the same concepts as the CARES or its subscales. These seven independent, but complementary, instruments are all considered to be international ‘gold standards’ or are frequently used instruments. These instruments were selected as they represent domains similar to the summary scales and global score of the CARES. All of them have been previously used in Belgian research. The concurrent validity of the original CARES was evaluated in comparison with the SCL-90, DAS and the KPS [34]. However, these instruments do not match the content of the CARES as completely as the set of concurrent measures in current study does. The concept equivalence and expected correlation with the CARES, to evaluate concurrent validity, is shown in Table 5.

**Table 5:** Expected correlations of concurrent measures with CARES summary scales and global score

Summary scale and CARES global score	Concurrent instrument	Expected correlation^a^
Physical	KPS	-
Psychosocial	HADS-A HADS-D	+
Psychosocial	SSL-I SSL-D	- +
Marital	MMQ-M	-
Sexual	MMQ-S	-
CARES Global score	EORTC-QLQ -C30	-
CARES Global score	DT	+
CARES Needs	Care Needs Questionnaire E. Pauwels	+

^a^ ‘-‘= negative correlation, ‘+’ = positive correlation

*Abbreviations CARES* Cancer Rehabilitation Evaluation System, *KPS* Karnofsky Performance status Scale, *HADS* Hospital Anxiety and Depression Scale, *SSL-I* Social Support List – Interactions, *SSL-D* Social Support List – Discrepancies, *MMQ-M* Maudsley Marital Questionnaire – Marital, *MMQ-S* Maudsley Marital Questionnaire – Sexual, *EORTC-QLQ -C30* European Organisation of Research and Treatment for Cancer Quality of Life Questionnaire Core 30, *DT* Distress Thermometer

#### CARES [31–37]

The original CARES contains 139 items; however, not all 139 items apply to all patients and therefore patients complete a minimum of 93 items or a maximum of 132 items. Patients can rate each item, formulated as problem statement, on a 5-point Likert scale with zero representing “not at all” (no problem) and four representing “very much” (severe problem). The clinical form of the instrument that will be used in this study allows a patient to indicate which problems they believe require help, ticking ‘yes’ or ‘no’ on the question ‘Do you want help?’. Scores for the five summary scales can be computed as well as a CARES global score and an average severity score.

#### Karnofsky Performance status Scale (KPS) [48, 54, 55]

The KPS is an 11-point scale to judge the physical and daily functioning of a patient and ranges from 0 (completely dependent, not able to care for oneself) to 100 (fully active, not dependent and capable of normal activity without limitations). This measure is currently used worldwide in research and practice and has been administered for many years. The KPS has got good psychometric properties (interrater reliability: *r* = .97; concurrent validity: *p* < .001; predictive validity: *r* = .30).

#### The Hospital Anxiety and Depression Scale (HADS) [56–58]

The HADS was developed to identify symptoms of anxiety and depression in medically ill patients, and is used extensively in cancer patients and had excellent psychometric qualities. The questionnaire contains 14 items with four possible answers with scores ranging from 0 to 3. Higher scores on the two subscales (each consisting of seven items) indicate a higher level of anxiety or depression and the total score of the HADS (score-ranges from 0 to 42) can be used as a global measure of psychological distress. The HADS has got good psychometric properties (internal consistency: α = .67–.93; PCA: two factor solution; concurrent validity: *r* = .49–.83; subscale inter-correlations: *r* = .40–.74).

#### The Social Support List – Interactions and Discrepancies (SSL-I and –D) [59–61]

The SSL is a questionnaire with 75 items, 41 on experienced social interaction and 34 on experienced social discrepancies. In the first part of the questionnaire participants indicate how frequently certain social interactions occur on a 4-point Likert scale from 1 (‘seldom or never’) to 4 (‘very often’), with higher scores representing higher levels of social support. A second part of the SLL indicates the social discrepancies participants experience ranging from 1 (‘I would like it to happen more often’) to 4 (‘it happens too often’). Higher scores on the SSL-D indicate a greater lack of social support. The psychometric properties of the SSL are positively evaluated (internal consistency: α = .53–.93, test-retest reliability: *r* = .62–.85).

#### The Maudsley Marital Questionnaire (MMQ) [62–65]

The MMQ contains three scales exploring Marital (10 items), Sexual (five items) and General Life (five items) adjustment. The respondent is asked to indicate an answer from a series of possible answers, on a scale ranging from 0 to 8. The wording of response categories differ for each item depending on nature of the question. The MMQ has good psychometric properties (internal consistency: α = ..66–.90; PCA: three factor solution; subscale inter-correlations: *r* = .33–.60).

#### The European Organisation of Research and Treatment for Cancer Quality of Life Questionnaire Core 30 (EORTC-QLQ-C30) [66]

The EORTC QLQ-C30 is an internationally validated and widely used cancer-targeted QOL instrument, incorporating five functional scales (physical, role, cognitive, emotional and social) and three symptom scales (fatigue, pain and nausea, and vomiting). Items are answered on a 4-point Likert scale from 1 (‘not at all’) to 4 (‘very much’). The last two items on global health and QOL have 8-point answering scales ranging from 1 (‘very poor’) to 7 (‘excellent’). The EORTC QLQ -C30 is subject of a many validation studies worldwide, generally concluding the questionnaire is a QOL instrument with good psychometric properties relevant to different cancer-patient populations (internal consistency: α = .52–.92; test-retest reliability: *r* = .72–.84; scale inter-correlations: *r* = -.69–.85; responsive to change of health status).

#### The Distress Thermometer (DT) together with a Problem List (PL) [67–69]

Patients are asked to rate their overall distress on a visual analogue scale (presented as a thermometer) from 0 (‘no distress’) to 10 (‘extreme distress’). The DT is accompanied by the PL, which includes 35 items that address five life domains (practical, family/social, emotional, spiritual, and physical problems). Participants indicate if the topics of the items poses problems for them. At the end of the survey people are asked if they want to talk to a professional about their problems. The DT is frequently used in clinical practice and research all over the globe, in combination with the PL. This has proved to have good internal consistency (α = .80–.90).

#### Care Needs questionnaire [70]

The Care Needs questionnaire was developed to assess the care needs of cancer survivors regarding relevant themes during reintegration: physical functioning, psychological functioning, self and body image, sexuality, relationship with partner, relationship with others and work and social security related aspects. For each theme, participants are asked whether they wish to receive information or support, in what way, when they prefer to receive information and support, and to what extent this need already has been met. Each of the questions are answered on 3- and 4-point Likert scales with different wording.

The *second questionnaire* contains a second CARES survey and specific supplementary questions to get data on participants’ experiences with the CARES. This second study component will be completed to assess test-retest and a patient-acceptability of the measure. Table 6 gives a detailed summary on the composition of both questionnaires and the measured concepts.

**Table 6:** Composition of questionnaires for quantitative data collection

Questionnaires	Instrument	Data collected
*Questionnaire 1*		
*T*_*0*_ *Baseline*	Self-administered questions on socio-demographic and medical aspects	Age, sex, marital status, children, education, employment status, household income, social surrounding, involved care providers, diagnosis, date of diagnosis, treatment(s), start and end dates of treatments.
	CARES	Quality of life and rehabilitation needs
	KPS	Physical and daily functioning
	HADS	Symptoms of anxiety and depression
	SSL	Social support
	MMQ	Marital and sexual life adjustment
	EORTC-QOL-C30	Quality of life
	DT + PL	Distress and problems
	Care needs questionnaire administered by E. Pauwels	Care needs
*Questionnaire 2*		
*T*_*1*_ *After 1 week*	CARES	Quality of life and rehabilitation needs
	Self-administered questions	Relevance of CARES-topics, timespan filling in, mode preference,…

*Abbreviations CARES* Cancer Rehabilitation Evaluation System, *KPS* Karnofsky Performance status Scale, *HADS* Hospital Anxiety and Depression Scale, *SSL* Social Support List, *MMQ* Maudsley Marital Questionnaire, *EORTC-QLQ -C30* European Organisation of Research and Treatment for Cancer Quality of Life Questionnaire Core 30, *DT* Distress Thermometer, *PL* Problem List

### Ethical considerations

All local ethical committees of the participating hospitals (Ethical Review Commission Jessaziekenhuis; Committee Medical Ethics Ziekenhuis Oost-Limburg; Ethical Committee AZ Vesalius; Ethical Committee Mariaziekenhuis Noord-Limburg) and the university (Medical Ethical Committee Hasselt University) reviewed all study materials including: the recruitment materials and procedure, informed consent form, the questionnaires and the overall study protocol. The leading ethical committee (ERC Jessaziekenhuis) coordinated the process, collected feedback and granted approval on 26^th^ of February 2014 (BE24320149544). The leading ethical committee also reviewed and approved study protocol amendments.

### Data analysis

The Statistical Package for Social Sciences (SPSS; Chicago, IL) version 22.0 will be used for statistical analyses of the quantitative data. A range of analyses are required to report the reliability and validity of the translated CARES version.

### Reliability

The reliability of the CARES will be evaluated by computing the *internal consistency* of summary scales, with the aim to find a Cohen’s Alpha of at least .70 [71, 72]. *Test-retest reliability* will be investigated by computing the intra-class correlations between the summary scale scores and total-CARES scores of the first and second CARES administration, requiring a correlation ≥ .70 [71, 73].

### Construct validity

The five factor structure as found in previous CARES-research will be examined with principal component analysis to evaluate construct validity. Following previous validation techniques applied in the original CARES development process, items and subscales with a factor loading higher as .30 are seen as loading on a factor [34, 35]. Confirmatory factor analysis will not be applied since sample size will be limited. As well inter-correlations of summary scales and the CARES Total will be explored. Moderate correlations between the subscales (*r* = |.30|-|.70|) and moderate to high correlations with the CARES Total (*r* ≥ .30) would support construct validity, since this would indicate that the subscales indeed measure distinct, but related concepts that contribute to the larger concept of QOL.

### Concurrent validity

Spearmans rank correlations will be computed to evaluate concurrent validity of the CARES global score and the summary scales with the seven concurrent instruments (Table 5). Correlations will be judged low, moderate and high, when their absolute values are respectively < .30, from .30 to .50 and ≥ .50 [74].

If the psychometric qualities do not show as expected, these will be studied in more detail with qualitative research data on CARES content and feasibility to search for explanations.

## Discussion

To achieve good quality care it is important to provide it as efficiently as possible, and adapted to the individual needs of every patient. A stepped care approach according to patients’ level of need could serve to tailor care efficiently and appropriately, however this necessitates reliable and valid screening and assessment tools to support clinicians in the identification of psychosocial concerns and care needs of their patients. The current English CARES is such an instrument. This paper describes a comprehensive protocol for translating and validating a Flemish CARES version. This addresses a critical gap in current clinical screening, and adds a tool to assess and improve the delivery of patient-centered care.

Unique in this study is the use of a wide range of comparative instruments to examine the concurrent validity of the CARES. Many other validation studies use only a few dimensions, not reflecting the whole concept of the specific instrument [28, 29, 75]. In contrast, this study will include an instrument to examine concurrent validity almost for each summary scale and the CARES global score. While our study is set up to examine the psychometric quality of the Flemish CARES, an additional advantage is that the use of several concurrent instruments will provide us with a wealth of data. The use of the KPS, HADS, SSL, MMQ, EORTC QLQ-C30, DT, PL and the Care needs questionnaire of Pauwels et. al. provides data on psychological, social, marital and sexual wellbeing, QOL, distress and care needs of patients treated for cancer. These can be used to explore potential relationships between mutual care-domains and with socio-demographic and medical characteristics. The recruitment for this study has begun (March 2014) and will continue until the end of 2014 or until the desired sample size is reached.

As in all studies, this study has some limitations. Firstly, the completeness of the CARES content to assess QOL and supportive care needs should be considered. In comparison to other psychometric positively evaluated needs assessment tools for cancer patients, like the Supportive Care Needs Survey (SCNS) [76], the CARES does not include items on spiritual and existential well-being [29]. However, on other domains of well-being, we can judge in favor of the CARES content. The content of the CARES matches with our thoughts about the bio-psychosocial impact of cancer on patients’ lives and possibly resulting care needs. Furthermore, the content validity, completeness and feasibility of the CARES for Flemish cancer patients will be explored in the qualitative part of the larger study combining qualitative and quantitative methods. If the results of the study described in this research protocol result in a negative evaluation of the CARES’ psychometric properties, or it appears from the focus group discussions that there are deficits in the CARES content, formulation of items, or feasibility, adjustments for an improved Flemish version will be made. We plan to use this ‘final’ Flemish version in a pilot study where it will support the routine assessment and management of patients’ psychosocial concerns and needs in a clinical pathway with medical and psychosocial components. In the future, the instrument will also be made available for use in clinical practice.

Secondly, the use of two procedures to invite patients to participate in the study can introduce bias. An invitation to participate in research from a member of the medical team or by post could result in different response rates in both subgroups. This is a demanding study for patients and therefore also for professionals to convince patients to participate. Hence, some flexibility in the process of patient recruitment is needed. Some departments prefer a personal approach and want to invite their patients for the research personally, while others do not find the time in the clinical appointment to do this. Both procedures have been previously used in other validation research [66, 75, 77–80]. To assess any recruitment or consent bias, we will compare the data from the group of patients invited to participate in the hospital to the group invited by post.

Thirdly, the questionnaire package composed with the CARES and several concurrent instruments asks for a time-investment of approximately an hour. This could present a burden to participants, resulting in discontinuation of participation. However, preliminary study results report approximately 58 % of the questionnaires distributed were returned completed. Eighty-four percent of the 153 participants who returned the first questionnaire also returned the second questionnaire completed.

Fourthly, the time between completing of the first and the second CARES survey could pose some problems. To examine test-retest reliability of an instrument the time period between two completions should be short enough to ensure that clinical change has not occurred, though long enough to prevent recall. While 1 or 2 weeks are recommended in literature [53], we ask participants to fill out the second questionnaire 1 week after the first. Preliminary results have shown some participants forget to fill in the second questionnaire or do not do so in a timely manner. They are reminded with a phone call by the researcher when de second questionnaire is not received in the recommended period of time. When data collection is completed, the time span between the two CARES-completions will be evaluated.

Fifthly, in earlier research the CARES was validated and used as a research tool for participants with various types of cancers, various cancer stages, at different phases of the care process, and often without age restrictions [31–44]. Because of the strict inclusion criteria we applied in our study, we have to state that the validation evidence from this study will not apply to patients above 60 years of age, and those with metastatic disease or in palliative care.”

## Conclusions

In summary, this study protocol describes a unique and thorough examination of the psychometric robustness of a QOL and needs assessment tool. Internal consistency of summary scales, test-retest reliability, content validity, feasibility, construct validity and concurrent validity of the Flemish CARES are explored. Likewise, the use of several concurrent instruments will provide insight in the QOL, physical, emotional, social, relational and sexual functioning and well-being, distress and care needs of the research population. We expect to find positive results on the reliability and validity of the Flemish CARES version. Comprehensive assessment with the CARES throughout the care trajectory can contribute to timely identification of cancer patients psychosocial concerns and care needs to refer them to tailored care and improve the quality of psychosocial cancer care.

## Aditional files

Additional file 1: Summary of needs assessment tools and psychometric properties. The table in this file displays a summary of 24 needs assessment tools and their psychometric properties. Items, domains, validity (content validity and other types of validity), reliability (internal consistency and reproducibility), responsiveness and feasibility are discussed. (DOCX 44 kb) Additional file 2: CARES Questionnaire. (PDF 7917 kb) Additional file 3: CARES Patient profile. (PDF 173 kb)

## Abbreviations

CARES: Cancer Rehabilitation Evaluation System
CARES-SF: Cancer Rehabilitation Evaluation System-Short Form
DAS: Dyadic Adjustment Scale
DT: Distress Thermometer
EORTC-QLQ-C30: European Organisation of Research and Treatment for Cancer Quality of Life Questionnaire Core 30
HADS: Hospital Anxiety and Depression Scale
KPS: Karnofsky Performance status Scale
MMQ: Maudsley Marital Questionnaire
PL: Problem List
PRO: Patient-Reported Outcome
QOL: Quality Of Life
SCL-90: Symptom Checklist-90
SCNC: Supportive Care Needs Survey
SPSS: Statistical Package for Social Sciences
SSL-I: Social Support List – Interactions
SSL-D: Social Support List – Discrepancies
