# Supplementary material for: Assessing cancer patients’ quality of life and supportive care needs: Translation-revalidation of the CARES in Flemish and exhaustive evaluation of concurrent validity
Source: BMC Health Serv Res. 2016 Mar 11;16:86. doi: 10.1186/s12913-016-1335-4 (PMC4788884; doi:10.1186/s12913-016-1335-4)
Supplement: Additional file 1: — Summary of needs assessment tools and psychometric properties. The table in this file displays a summary of 24 needs assessment tools and their psychometric properties. Items, domains, validity (content validity and other types of validity), reliability (internal consistency and reproducibility), responsiveness and feasibility are discussed. (DOCX 44 kb) [file 12913_2016_1335_MOESM1_ESM.docx]

Table 1bis Summary of needs assessment tools and their psychometric properties

| **Instrument** | **Items and domains** | **Validity** | | **Reliability** | | **Responsiveness** | **Feasibility** |
| --- | --- | --- | --- | --- | --- | --- | --- |
|  |  | **Content Validity** | **Other types of validity** | **Internal consistency** | **Reproducibility** |  |  |
| **CaNDI**  Cancer Needs Distress Inventory | 39 items;  7 domains: depression, anxiety, emotional, social, health care, practical, physical | Derived from pool of items of concerns of cancer patients.  Literature review.  Revised in 2005, focus groups with patients and psycho-oncology professionals. | Good Spearman’s r of total score with HADS, FACT-G, BSI and PDS.  Good Spearman’s r of CaNDI anxiety and depression with BSI anxiety and BSI depression.  Lack sufficient power to adequately test the factor structure of the CaNDI  Not all subscales validated. | All α >0.70  Time 1: 0.91 for full and retest  Time 2: 0.92 for retest sample | Completed 2nd CaNDI 3 to 7 days  ICCs ≥ 0.99 | - | Time: N/A;  Reading level: 5.5 reading grade level;  Acceptability: N/A. |
| **CARES**  Cancer Rehabilitation Evaluation System | 93-132 items;  31 subscales taken together in 6 domains: physical, psychological, medical interaction, marital, sexual, miscellaneous | Literature.  Interviews with patients & family. Expert review. | Factor-analysis resulted in 5-factor solution.  Concurrent validity with SCL-90, KPS, DAS and visual analogue scale QOL. Good agreement with interviewers.  Discriminant validity: able to distinguish patients with different disease stages. | Domains α ranged from .87 to .94 | Subscales and CARES-Total: r= .84 - .95  87% agreement n=71,  time=1 week | - | Time: 20 min (range10-45);  Reading level:  N/A;  Acceptability: most found it easy to use. |
| **CARES-SF**  Cancer Rehabilitation Evaluation System-Short Form | 38-57 items;  5 domains: physical, psychological, medical interaction, marital, sexual | Selected from the CARES by experts. | Factor-analysis resulted in 5 factor solution.  Concurrent validity with CARES, FLIC, KPS, DAS. Large sample sizes. | Domains α ranged from .60 to .84 | Dimensions:  r=.69 - .92  81%-86% agreement n=120, time=10 days | Find physical, psychosocial change with time. Correlated with FLIC @ 1, 7, 14 months post-diagnosis | Time: on average 10min;  Reading level: N/A;  Acceptability: N/A. |
| **CCM**  Cancer care monitor | 38 items;  6 domains: general physical symptoms, treatment side effects, acute distress, despair, impaired ambulation, impaired performance (plus one global QOL-index) | Literature.  Physician Judgements.  Review by professionals  and patients. | Convergent and divergent validity through comparison with BSI, SF-36, MSAS, LSI, SWLS. | Domains ranged from  α= 0.80 to α= 0.89. | Time: between 1  and 7 days apart  (correlations ranged from r=0.90 to 0.74)  Time: between 8 and 14 days apart (correlations ranged from r=0.87 to 0.74). | - | Time: 20 min to  complete paper  version, 12 min to complete electronic version;  Reading level: 85%, completed high school or greater education;  Acceptability: patients expressed a strong preference for the electronic form (versus  the paper form). |
| **CHOICEs** assessment  Creating better health outcomes by improving communication about patients’ experiences assessment | 112 items;  6 domains: Cancer  specific symptoms, functional problems, physical, psychosocial, emotional, spiritual  (plus 2 global  ratings: health  and QOL) | Literature.  Review by an expert  focus group specialists in cancer care.  Review by patients. | - | “Ease of Use”: α= 0.98  “Satisfaction”: α= 0.86 | - | - | Time: median 9 min (range=0.5  to 49 min); 25%  percent of the sample used ≤5 min;  Reading level: N/A;  Acceptability: (a) ease of use: 80% without  assistance, 20% some assistance (weak, disability,  convenience).  Positive overall ‘Ease of Use’ score=5.06  (range −16 to +16) (b)  Satisfaction: scores positively skewed in both groups. |
| **Concerns** **checklist** | Refined version:12items,  original source:53 items;  3 domains: illness, practical, psychological | Literature.  Retrospective study data.  Pilot work.  Review by patients. | Factor analysis resulted in 3-factor solution. | - | - | - | Time: N/A;  Reading level:  N/A; Acceptability: N/A. |
| **CNAT**  Comprehensive needs assessment tool in cancer | 59 items;  8 domains: iInformation, psychological, health care staff, physical symptoms, hospital services, family/interpersonal, spiritual/religious, social. | Review of existing tools.  Patient interviews.  Patients and health professionals identified relevant items  Pilot testing with 15 patients. | Exploratory factor analysis: 7 factor structure (64.2% variance).  Convergent validity: low to moderate Spearman r with EQ5D. | All α >0.70 total scale α =0.97; subscales: α =0.80 to 0.97 | - | - | Time: N/A;  Reading level:  N/A; Acceptability: N/A. |
| **CNQ**-**SF**  Cancer Needs Questionnaire Short Form | 32 items;  5 domains: psychological, health information, physical and daily living, patient care and support, interpersonal communication. | From original CNQ | Factor analysis resulted in 5 factors (68% of variance).  Good correlation with EORTC QLQC-30 and BDI. | Domains ranged from α =0.77 to α =0.99. | - | - | Time: 20 min;  Reading level: 4th or 5th grade; 25% non-completion rate;  Acceptability: N/A. |
| **CPILS**  Cancer Problems in  Living Scale | 31 items;  4 domains: physical distress, emotional distress, employment/financial problems, fear of recurrence. | Patient interviews.  Patient surveys.  Clinical opinion. | Exploratory Factor analysis resulted in 4 factors.  Convergent validity:  Physical correlated with RSCL-M (r=.50) and SF-36 (r= -.31 to -.45)  Emotional correlated with POMS-SF (r=.27 to .38) and SF-36 (r= -.18 to -.31) | All α >0.70  Physical α =0.84  Emotional α =0.87  Financial α =0.78  Fear of recurrence α =0.84. | - | - | Time: N/A;  Reading level:  N/A; Acceptability: N/A. |
| **CPNS**  Cancer Patient Need Survey | 51 items;  5 domains: coping, help, information, work, and cancer shock | Interviews with nurses, patients, & caregivers using. Objective Content Test & Q-sort method. | - | Overall α= 0.91  Importance α: .83-.93  How well met α: .79-.95  Domains ranged from α= .88 to α= .92 | - | - | Time: 2-45 min;  Reading level N/A; Acceptability: reported no problems when used. |
| **CPNQ**  Cancer Patient Need Questionnaire | 71 items;  5 domains: psychological needs, health info, ADLs,  patient care/support, interpersonal communication. | Literature. Interviews.  Expert review.  Pilot test. | Discriminant validity: able to distinguish patients with different disease stages. | Domains α ranged from .78 to .90 | Intercorrelation all significant kappa > .4  n=124,  time=10-14 days | - | Time: 20 min;  Reading level: 4^th^ or 5^th^ grade; Acceptability: 25% non-completion rate. |
| **Distress** **management** **tool** | 36 items;  5 domains: practical, family, emotional, spiritual/  religious, physical  (plus 1 general  distress item). | Literature.  Expert review- NCCN panel. | - | - | - | - | Time: N/A;  Reading level: N/A; Acceptability: N/A. |
| **INM**  Information Needs Measure | 9 information categories | Literature.  Based on works by Derdiarian.  Expert review. | - | Kendall zeta: .95-.99.  Kendall coefficient of agreement: .20-.35. | - | - | Time: N/A;  Reading level: N/A;  Acceptability: N/A. |
| **NEQ**  Need Evaluation Questionnaire | 23 items;  4 domains: information  regarding  diagnosis/prognosis,  examination/treatment, communication, relational, (plus 12  additional items) | Interviews.  Pilot tests. | Factor analysis on the scale only partially confirms the hypothesized  structure. Later study demonstrated good fit. | Domains: α ranged from .69-.81 | Cohen’s kappa ranged from .54-.94  Time=1week | - | Time: 5 min; Reading level: N/A;  Acceptability: 63% of patients OK; 24% in-complete;  3% missing data. |
| **OCPC**  Oncology Clinic Patient Checklist | 86 Items;  15 domains:information, fatigue, pain, nutrition, speech and  Language, respiration, bowel and  bladder, transportation, mobility, self and home  care, vocational  and educational, interests and  activities, family, interpersonal  relationships, Emotional, (plus 3 open-ended  questions) | Data from previous  research.  Based on items from  other tool. | - | - | - | - | Time: N/A;  Reading level: N/A  Acceptability: Checklist  was accepted for  practical use—process evaluation by staff (100% response rate)  and patients 78%)  (after 4 months);  positive response from nursing staff. Usefulness, 82% (pilot work, n=11 patients) |
| **PINQ**  Patient Information Need Questionnaire | 17 items;  2 domains: disease-oriented and information about access to help & solution | Literature.  Interviews. | Correlated with RSC, State-Anxiety Inventory & MMPI D-scale. | Domains ranged from α= .88 to α= .92;  Inter-item correlation >0.2 | - | Detected the changing needs of patients at three time points before and after first treatment | Time: N/A; Reading level:N/A Acceptability: reasons to refuse: not wanting to be reminded of their illness, feeling too old, etc. |
| **PNAS**  Psychosocial needs assessment survey | 34 items;  4 domains: informational, practical, supportive, spiritual. | Literature review.  Clinical opinion. | - | No data on construct validity.  Kuder-Richardson 20 statistic: Information: 0.90, Practical: 0.86, Supportive: 0.83, Spiritual: 0.90.  Subscale correlations:  r=.57 to .82 | - | - | Time: N/A;  Reading level: N/A; Acceptability: N/A. |
| **PNAT**  Patient Needs Assessment Tool | 16 items;  3 domains: physical, psychological, and social | Literature.  Clinical experience. | Physical domain correlates with KPS; Psychological with GAIS, BSI MPAS, BDI Social with ISEL. | Domains ranged from α= .85 to α= .94 | Interrater reliability: Friedman: .87, .76, .73;  Spearman rank order: .59- .98 | - | Time: 20-30 min.;  Reading level: N/A;  Acceptability: N/A. |
| **PNI**  Psychosocial Needs Inventory | 48 items;  7 domains: related to health professionals, information needs, related to support networks, identify needs, emotional and spiritual, practical and childcare need. | Literature.  Interviews.  Focus group. | Discriminant validity: detected the differences among needs at four critical movements of cancer trajectory. | α > .70 for each of the first six domains. | - | - | Time: N/A; Reading level: N/A; Acceptability: 59% non-response rate and the characteristic of the non-respondents was examined. |
| **Problem** **checklist** | 16 items;  4 domains: daily living, relationships, economics, emotions, (plus 2 other) | Literature.  Audit data.  Research study (n=505). | Factor analysis endorsed the 4-factor structure  (accounting for 64% of variance) with the components on Economics and Emotions being particularly credible | Domains ranged from  α= 0.70 to 0.82. | - | - | Time: N/A;  Reading level: N/A;  Acceptability: patients found it quick and easy to complete. |
| **SCNS**  Supportive Care Needs Survey | 61 items;  5 domains:  psychological needs, health information, physical/daily living needs, patient care & support, and sexuality | Based on CPNQ. .  Expert review.  Pilot test. | - | Domains ranged from α= .87 to α= .97. | - | - | Time: 20 min;  Reading level: 5^th^ grade; Acceptability: patients found it understandable, 35% non-completion. |
| **SCNS**-**SF34**  Supportive Care Needs Survey Short Form | 34 items;  5 domains:  psychological needs, health information, physical/daily living needs, patient care & support, and sexuality | Selected from original SCNS. 20 items factor loading >0.70  6 items: item-to-total correlation > domain cut-point & factor loading 0.51–0.69.  4 items factor loading 0.64–0.74 and clinically important  4 items clinically important | Confirmatory factor analysis (CFA) of five factors (73% of the total variance)  Known-groups validity: remission vs no remission patient using summated domain mean score. Patients not in remission had higher scores.  Convergent validity:  Correlated good with DT, HADS anxiety, HADS depression and QLQ-C30 global. | All α>0.70 (α=0.86 to 0.96)  Item-to-total score correlation coefficients r>0.55 | - | - | Time: N/A;  Reading level:  Flesch–Kincaid Grade Level 7.2;  Acceptability: N/A. |
| **SNST**  Supportive Care Needs screening Tool | 40 items;  5 domains: physical, social, psychological, information, spiritual. | Original pool 340 items taken from 20 existing tools. Expert opinion to reduce items.  Pilot test  Patient interviews. | - | - | - | - | Time: N/A;  Reading level: N/A;  Acceptable to patients and staff  Usability high for staff. |
| **Symptoms** **and** **concerns** **checklist** | 29-32 items;  4 domains: physical  symptoms, cognitive/  psychological, other  concerns, patient  defined | Literature.  Expert panel.  Pilot work.  Patient.  Interviews. | Generally demonstrated  convergent validity  when compared  with SDS, POS.  Able to discriminate between different groups of patients (e.g. Outpatients vs.  hospital inpatients). | Overall: α = .85 | Time: over 2  consecutive days—  weighted Kappa  0.35–0.77 |  | Time: 5 min;  Reading level: N/A;  Acceptability: 97% felt  comprehensive, 82% felt easy to complete, 79% good idea, 98%  participated, 7%  completed all items. |
| *Abbreviations: HADS (Hospital Anxiety and Depression Scale), FACT-*G (Functional Assessment of Cancer Therapy – General), *PDS* (Paulhus Deception Scales),SCL-90 (Symptom Checklist-90), DAS (Dyadic Adjustment Scale), KPS (Karnofsky Performance Status), FLIC ( Functional Living Index-Cancer),BSI (The brief symptom inventory), SF-36 (The Short Form (36) Health Survey), MSAS (The memorial symptom assessment scale), LSI (The lifesatisfaction index–short form), SWLS (The satisfaction with life scale), EQ5D (EuroQOL five dimensions questionnaire), EORTC QLQC-30 (European Organisation of Research and Treatment for Cancer Quality of Life Questionnaire Core 30), RSCL-M (Rotterdam Symptom checklist-Modified), POMS-SF (Profile Of Mood States-Short Form), NCCN (National Comprehensive Cancer Network), RSC (Rotterdam Symptom Checklist), MMPI-D (Minnesota Multiphasic Personality Inventory-Depression), GAIS (Global Adjustment to Illness Scale), MPAS (Memorial Pain Assessment Scale), BDI (Beck Depression Inventory), ISEL (Interpersonal Support EvaluationList ), PNI (Psychosocial Needs Inventory), SDS (Symptom Distress Scale), POS (Palliative care Outcome Scale). | | | | | | | |
