# Supplementary material for: Assessing cancer patients’ quality of life and supportive care needs: Translation-revalidation of the CARES in Flemish and exhaustive evaluation of concurrent validity
Source: BMC Health Serv Res. 2016 Mar 11;16:86. doi: 10.1186/s12913-016-1335-4 (PMC4788884; doi:10.1186/s12913-016-1335-4)
Supplement: Additional file 2: — CARES Questionnaire. (PDF 7917 kb) [file 12913_2016_1335_MOESM2_ESM.pdf]

CARES
CAncer Rehabilitation Evaluation System

Developed
by
C. Anne Coscarelli Schag, Ph.D.
and
Richard L. Heinrich, M.D.

Copyright © CARES Consultants, 1988

CARES
CAncer Rehabilitation Evaluation System

Patient Information

Name: ____________________ ID #: ____________

Date: ____________

Age: ____

Sex: M F

Type of Cancer: ____________________

Date of Diagnosis: ____________ Name of Physician: ____________

Instructions

Below is a list of Problem Statements that describe situations and experiences of individuals who have or have had cancer. Read each statement and circle the number that best describes **HOW MUCH EACH STATEMENT APPLIES TO YOU** during the **PAST MONTH, INCLUDING TODAY**. Some sections will not apply to you. Please skip these sections and proceed to the next one as directed. For any problem statement that you rate between 1 and 4, indicate whether this is a problem with which you would like help by circling *Y* for *yes* or *N* for *no.*

Example

**How much does it apply to you?**	Not at all	A little	A fair amount	Much	Very much	Do you want help?
1. I have difficulty walking	0	(1)	2	3	4	*Y* (*N*)
2. I find that food tastes bad	0	1	2	3	(4)	(*Y*) *N*

© CARES Consultants, 2118 Wilshire Blvd, Suite 359, Santa Monica, California 90403 (310) 452-4152

How much does it apply to you?	Not at all	A little	A fair amount	Much	Very much	Do you want help?	
1. I have difficulty bending or lifting	0	1	2	3	4	Y	N
2. I have difficulty walking and/or moving around	0	1	2	3	4	Y	N
3. I have difficulty doing physical activities such as running and playing sports	0	1	2	3	4	Y	N
4. I do not have the energy I used to	0	1	2	3	4	Y	N
5. I have difficulty driving	0	1	2	3	4	Y	N
6. I have difficulty doing household chores	0	1	2	3	4	Y	N
7. I have difficulty bathing, brushing my teeth, or grooming myself	0	1	2	3	4	Y	N
8. I have difficulty preparing meals	0	1	2	3	4	Y	N
9. I am not interested in recreational activities like I used to be	0	1	2	3	4	Y	N
10. I do not engage in the recreational activities that I used to	0	1	2	3	4	Y	N
11. I do not have enough enjoyable activities to fill the day	0	1	2	3	4	Y	N
12. I have difficulty planning activities because of the cancer or its treatments	0	1	2	3	4	Y	N
13. I cannot gain weight	0	1	2	3	4	Y	N
14. I am continuing to lose weight	0	1	2	3	4	Y	N
15. I find food unappealing	0	1	2	3	4	Y	N
16. I find that food tastes bad	0	1	2	3	4	Y	N
17. I find it difficult to swallow	0	1	2	3	4	Y	N
18. I find that the cancer or its treatments keep me from working	0	1	2	3	4	Y	N
19. I find that cancer or its treatments interfere with my ability to work	0	1	2	3	4	Y	N
20. I frequently have pain	0	1	2	3	4	Y	N
21. I have chronic pain from scars and surgery	0	1	2	3	4	Y	N
22. I have pain that is not controlled by pain medication	0	1	2	3	4	Y	N

How much does it apply to you?	Not at all	A little	A fair amount	Much	Very much	Do you want help?	
23. I have pain that is controlled by pain medication	0	1	2	3	4	Y	N
24. I find that my clothes do not look good on me	0	1	2	3	4	Y	N
25. I find that my clothes do not fit	0	1	2	3	4	Y	N
26. I have difficulty finding clothes to fit	0	1	2	3	4	Y	N
27. I find that the medical team withholds information from me about the cancer	0	1	2	3	4	Y	N
28. I find that doctors don't explain what they are doing to me	0	1	2	3	4	Y	N
29. I find that nurses don't explain what they are doing to me	0	1	2	3	4	Y	N
30. I have difficulty asking doctors questions	0	1	2	3	4	Y	N
31. I have difficulty asking nurses questions	0	1	2	3	4	Y	N
32. I have difficulty expressing my feelings to the doctors and nurses	0	1	2	3	4	Y	N
33. I have difficulty telling my doctor about new symptoms	0	1	2	3	4	Y	N
34. I have difficulty understanding what the doctors tell me about the cancer or its treatments	0	1	2	3	4	Y	N
35. I have difficulty understanding what the nurses tell me about the cancer or its treatments	0	1	2	3	4	Y	N
36. I would like to have more control over what the doctors do to me	0	1	2	3	4	Y	N
37. I would like to have more control over what the nurses do to me	0	1	2	3	4	Y	N
38. I am embarrassed to show my body to others because of my illness	0	1	2	3	4	Y	N
39. I am uncomfortable showing my scars to others	0	1	2	3	4	Y	N
40. I am uncomfortable with the changes in my body	0	1	2	3	4	Y	N
41. I frequently feel anxious	0	1	2	3	4	Y	N
42. I frequently feel depressed	0	1	2	3	4	Y	N
43. I frequently feel angry	0	1	2	3	4	Y	N

How much does it apply to you?	Not at all	A little	A fair amount	Much	Very much	Do you want help?	
44. I frequently feel upset	0	1	2	3	4	Y	N
45. I frequently feel overwhelmed by my emotions and feelings about the cancer	0	1	2	3	4	Y	N
46. I have difficulty sleeping	0	1	2	3	4	Y	N
47. I have difficulty concentrating	0	1	2	3	4	Y	N
48. I have difficulty remembering things	0	1	2	3	4	Y	N
49. I have difficulty thinking clearly	0	1	2	3	4	Y	N
50. I have difficulty telling my friends or relatives to come over less often	0	1	2	3	4	Y	N
51. I have difficulty telling my friends or relatives to leave when I do not feel well	0	1	2	3	4	Y	N
52. I have difficulty asking my friends or relatives to do something fun with me	0	1	2	3	4	Y	N
53. I do not know what to say to my friends or relatives	0	1	2	3	4	Y	N
54. I have difficulty asking friends or relatives to do things for me	0	1	2	3	4	Y	N
55. I have difficulty telling my friends or relatives about the cancer	0	1	2	3	4	Y	N
56. I have difficulty asking my friends or relatives to come over more often	0	1	2	3	4	Y	N
57. I find that my friends or relatives tell me I'm looking well when I'm not	0	1	2	3	4	Y	N
58. I find that my friends or relatives withhold information from me	0	1	2	3	4	Y	N
59. I find that my friends or relatives avoid talking with me about the cancer	0	1	2	3	4	Y	N
60. I find that my friends or relatives do not visit often enough	0	1	2	3	4	Y	N
61. I find that my friends or relatives do not call often enough	0	1	2	3	4	Y	N

How much does it apply to you?	Not at all	A little	A fair amount	Much	Very much	Do you want help?	
62. I find that my friends or relatives are uncomfortable when they visit me	0	1	2	3	4	Y	N
63. I find that friends or relatives have difficulty talking with me about my illness	0	1	2	3	4	Y	N
64. I feel uncomfortable when I see other patients getting treatments	0	1	2	3	4	Y	N
65. I become nervous when I have to go to the hospital	0	1	2	3	4	Y	N
66. I become nervous when I am waiting to see the doctor	0	1	2	3	4	Y	N
67. I become nervous when I am waiting to find out the results of tests	0	1	2	3	4	Y	N
68. I become nervous when I am having diagnostic tests	0	1	2	3	4	Y	N
69. I become nervous when I get my blood drawn	0	1	2	3	4	Y	N
70. I worry about whether my treatments are working	0	1	2	3	4	Y	N
71. I worry about whether the cancer is progressing	0	1	2	3	4	Y	N
72. I worry about not being able to care for myself	0	1	2	3	4	Y	N
73. I worry about how my family will manage if I die	0	1	2	3	4	Y	N
74. I do not feel sexually attractive	0	1	2	3	4	Y	N
75. I do not think my partner(s) finds me sexually attractive	0	1	2	3	4	Y	N
76. I am not interested in having sex	0	1	2	3	4	Y	N
77. I do not think that my partner(s) is interested in having sex with me	0	1	2	3	4	Y	N
78. I sometimes don't show up for my doctor's appointment	0	1	2	3	4	Y	N
79. I sometimes don't show up for my treatments	0	1	2	3	4	Y	N
80. I sometimes don't take my medication as prescribed	0	1	2	3	4	Y	N
81. I sometimes don't follow my doctor's instructions	0	1	2	3	4	Y	N
82. I have financial problems	0	1	2	3	4	Y	N

How much does it apply to you?	Not at all	A little	A fair amount	Much	Very much	Do you want help?	
83. I have insurance problems	0	1	2	3	4	Y	N
84. I have difficulty with transportation to and from my medical appointments and/or other places	0	1	2	3	4	Y	N
85. I am gaining too much weight	0	1	2	3	4	Y	N
86. I find some diagnostic procedures extremely painful	0	1	2	3	4	Y	N
87. I have frequent episodes of diarrhea	0	1	2	3	4	Y	N
88. I have times when I do not have control of my bladder	0	1	2	3	4	Y	N

Do you have children? **Yes** **No**

If No, skip to next section.

89. I have difficulty taking care of the children and/or the grandchildren	0	1	2	3	4	Y	N
90. I have difficulty helping my children cope with my illness	0	1	2	3	4	Y	N
91. I have difficulty helping my children talk about my illness	0	1	2	3	4	Y	N

Are you working or have you been been employed during the last month? **Yes** **No**

If No, skip to next section.

92. I have difficulty talking to my boss about the cancer	0	1	2	3	4	Y	N
93. I have difficulty talking to the people who work with me about the cancer	0	1	2	3	4	Y	N
94. I have difficulty telling my employer that I cannot do something because of my illness	0	1	2	3	4	Y	N
95. I have difficulty asking for time off from work for medical treatments	0	1	2	3	4	Y	N
96. I am worried about being fired	0	1	2	3	4	Y	N

How much does it apply to you?	Not at all	A little	A fair amount	Much	Very much	Do you want help?
**Did you look for work during the past month?** **Yes No**						
If No, skip to next section.						
97. I have difficulty finding a new job since I have had cancer	0	1	2	3	4	Y N
98. I find that employers are reluctant to hire people with a cancer history	0	1	2	3	4	Y N
**Have you been sexually active since your cancer diagnosis?** **Yes No**						
If No, skip to next section.						
99. I find that the frequency of sexual activity has decreased	0	1	2	3	4	Y N
100. I have difficulty becoming sexually aroused	0	1	2	3	4	Y N
101**a.** I have difficulty getting or maintaining an erection (**Males**)	0	1	2	3	4	Y N
**b.** I have difficulty getting lubricated (**Females**)						
102. I have difficulty reaching orgasm	0	1	2	3	4	Y N
**Are you married or in a significant relationship?** **Yes No**						
If No, skip to next section.						
103. My partner and I have difficulty talking about our feelings	0	1	2	3	4	Y N
104. My partner and I have difficulty talking about our fears	0	1	2	3	4	Y N
105. My partner and I have difficulty talking about what will happen after my death	0	1	2	3	4	Y N
106. My partner and I have difficulty talking about our future	0	1	2	3	4	Y N
107. My partner and I have difficulty talking about the cancer and what might happen	0	1	2	3	4	Y N

How much does it apply to you?	Not at all	A little	A fair amount	Much	Very much	Do you want help?
108. My partner and I have difficulty talking about wills and financial arrangements	0	1	2	3	4	Y N
109. I do not feel like embracing, kissing, or caressing my partner	0	1	2	3	4	Y N
110. My partner does not feel like embracing, kissing or caressing me	0	1	2	3	4	Y N
111. I am not interested in touching my partner	0	1	2	3	4	Y N
112. My partner is not interested in touching me	0	1	2	3	4	Y N
113. My partner and I are not getting along as well as we usually do	0	1	2	3	4	Y N
114. My partner and I are upset with each other more often than usual	0	1	2	3	4	Y N
115. My partner and I have so much time together that we get on each other's nerves	0	1	2	3	4	Y N
116. My partner and I are more distant than usual	0	1	2	3	4	Y N
117. My partner won't let me do activities that I am capable of doing	0	1	2	3	4	Y N
118. My partner spends too much time taking care of me	0	1	2	3	4	Y N
119. My partner does not take care of me enough	0	1	2	3	4	Y N
120. I have difficulty asking my partner to take care of me	0	1	2	3	4	Y N

Are you single and not in a significant relationship? **Yes** **No**

If No, skip to next section.

121. I have difficulty initiating contact with potential dates	0	1	2	3	4	Y N
122. I have difficulty meeting potential dates	0	1	2	3	4	Y N
123. I am afraid to go to places that I used to visit to meet dates	0	1	2	3	4	Y N
124. I have difficulty telling a date about the cancer or its treatments	0	1	2	3	4	Y N
125. I am afraid to initiate a sexual relationship with someone	0	1	2	3	4	Y N

How much does it apply to you?	Not at all	A little	A fair amount	Much	Very much	Do you want help?	
**Have you had chemotherapy treatments in the last month?** Yes No<br>***If No, skip to next section.***							
126. I become nervous when I get chemotherapy	0	1	2	3	4	Y	N
127. I become nauseated during and/or before chemotherapy	0	1	2	3	4	Y	N
128. I vomit during and/or before chemotherapy	0	1	2	3	4	Y	N
129. I feel sick when I think about my chemotherapy	0	1	2	3	4	Y	N
130. I feel nauseated after I receive chemotherapy	0	1	2	3	4	Y	N
131. I vomit after chemotherapy	0	1	2	3	4	Y	N
132. I feel tired after my chemotherapy	0	1	2	3	4	Y	N
133. I have other side effects after chemotherapy	0	1	2	3	4	Y	N
134. I have lost my hair and/or it is growing back slowly because of chemotherapy	0	1	2	3	4	Y	N
**Have you had radiation therapy treatments in the last month?** Yes No<br>***If No, skip to next section.***							
135. I feel fatigued after my radiation treatments	0	1	2	3	4	Y	N
136. I get nervous when I get radiation treatments	0	1	2	3	4	Y	N
137. I feel nauseous or vomit after my radiation treatments	0	1	2	3	4	Y	N
**Do you have an ostomy?** Yes No<br>***If No, skip to next section.***							
138. I have problems with ostomy care and maintenance	0	1	2	3	4	Y	N

How much does it apply to you?	Not at all	A little	A fair amount	Much	Very much	Do you want help?
**Do you have a prosthesis?** **Yes No**<br>***If No, skip to next section.***						
139. I have difficulty with my prosthetic device (artificial limb, breast prosthesis, etc.)	0	1	2	3	4	Y N

Please list any additional cancer or treatment–related problems that may not have been addressed:

A. ______________________________

B. ______________________________

C. ______________________________

D. ______________________________

E. ______________________________
