# Supplementary material for: Assessing cancer patients’ quality of life and supportive care needs: Translation-revalidation of the CARES in Flemish and exhaustive evaluation of concurrent validity
Source: BMC Health Serv Res. 2016 Mar 11;16:86. doi: 10.1186/s12913-016-1335-4 (PMC4788884; doi:10.1186/s12913-016-1335-4)
Supplement: Additional file 3: — CARES Patient profile. (PDF 173 kb) [file 12913_2016_1335_MOESM3_ESM.pdf]

# CAncer Rehabilitation Evaluation System (CARES): Patient Profile

**Patient Information**

Name: ______________________________

Date: ________

**Please Circle Normative Sample Used:**

1. Female Breast Cancer Norm
2. Female Other than Breast Cancer Norm
3. Female Combined Cancer Norm
4. Male Prostate Cancer Norm
5. Male Other than Prostate Cancer Norm
6. Male Combined Cancer Norm

| T-Score | CARES Global Score | CARES # Prob Endorsed | CARES Aver Sever | PHYSICAL Global Score | PSYCHOSOCIAL Global Score | MEDICAL INTERACTION Global Score | MARITAL Global Score | SEXUAL Global Score | % Rank |
|---|---|---|---|---|---|---|---|---|---|
| 80 | | | | | | | | | |
| 75 | | | | | | | | | 99 |
| 70 | | | | | | | | | 98 |
| 65 | | | | | | | | | 93 |
| 60 | | | | | | | | | 84 |
| 55 | | | | | | | | | 70 |
| 50 | | | | | | | | | 50 |
| 45 | | | | | | | | | 30 |
| 40 | | | | | | | | | 16 |
| 35 | | | | | | | | | 07 |
| 30 | | | | | | | | | 02 |
| 25 | | | | | | | | | |
| 20 | | | | | | | | | |

| | CARES Global Score | CARES # Prob Endorsed | CARES Aver Sever | PHYSICAL Global Score | PSYCHOSOCIAL Global Score | MEDICAL INTERACTION Global Score | MARITAL Global Score | SEXUAL Global Score |
|---|---|---|---|---|---|---|---|---|
| T | | | | | | | | |
| Raw | | | | | | | | |

COPYRIGHT © 1988 CARES Consultants

## PHYSICAL

### Ambulation
____ 1. diff bend or lift
____ 2. diff walk/move around
____ 3. diff do physical activ.
____ 4. reduction in energy

### Activities of Daily Living
____ 5. diff driving
____ 6. diff household chores
____ 7. diff bathe, brush groom
____ 8. diff prepare meals

### Recreational Activities
____ 9. no interest recreat activ
____ 10. not engage recreat activ
____ 11. not enough enjoyable activ
____ 12. diff planning activ

### Weight Loss
____ 13. cannot gain weight
____ 14. continue to lose weight
____ 15. food unappealing
____ 16. food tastes bad
____ 17. diff swallowing

### Difficulty Working
____ 18. cancer prevents work
____ 19. cancer interferes work

### Pain
____ 20. frequently has pain
____ 21. chronic pain scars/surgery
____ 22. pain not controlled medication
____ 23. pain controlled medication

### Clothing
____ 24. clothes not look good
____ 25. clothes not fit
____ 26. diff find clothes
____ *SUM* ___ *#(1-4) 26 # Potential*

## MEDICAL INTERACTION

### Problems Obtaining Info from Medical Team
____ 27. medical team withholds info
____ 28. doctors don't explain what do
____ 29. nurses don't explain what do

### Difficulty Communicating with Medical Team
____ 30. diff ask doctors questions
____ 31. diff ask nurses questions
____ 32. diff express feelings MD/RN
____ 33. diff tell doctor new symptoms
____ 34. diff understand MD about cancer
____ 35. diff understand RN about cancer

### Control of Medical Team
____ 36. wants more control over MD
____ 37. wants more control over RN
____ *SUM* ___ *#(1-4) 11 # Potential*

## *MARITAL

### Communication with Partner
____ 103. diff talk feelings
____ 104. diff talk fears
____ 105. diff talk happen after death
____ 106. diff talk future
____ 107. diff talk cancer
____ 108. diff talk wills/financial matters

### Affection with Partner
____ 109. doesn't feel like embrace,etc
____ 110. partner no feel like embrace, etc.
____ 111. no interest in touch partner
____ 112. partner no interest in touch

### Interaction with Partner
____ 113. not get along as well usual
____ 114. upset with other more often
____ 115. so much time together, on nerves
____ 116. more distant than usual

### Overprotection by Partner
____ 117. partner not let do activ capable of
____ 118. partner provides too much care

### Neglect of Care by Partner
____ 119. partner takes too little care
____ 120. diff ask partner to take care
____ *SUM* ___ *#(1-4) 18,0*
\# Potential Circle

## PSYCHOSOCIAL

### Body Image
____ 38. embarrassed to show body
____ 39. uncomfor show scars
____ 40. uncomfor with body changes

### Psychological Distress
____ 41. frequently anxious
____ 42. frequently depressed
____ 43. frequently angry
____ 44. frequently upset
____ 45. frequently overwhelmed by cancer
____ 46. diff sleep

### Cognitive Problems
____ 47. diff concentrating
____ 48. diff remembering
____ 49. diff thinking clearly

### Difficulty Communicat with Friends/Relatives
____ 50. diff tell frnd/rel to come less often
____ 51. diff tell frnd/rel to leave when not well
____ 52. diff ask frnd/rel to do fun things
____ 53. don't know what to say to frnd/rel
____ 54. diff ask frnd/rel help
____ 55. diff tell frnd/rel about cancer
____ 56. diff ask frnd/rel to come more

### Friends/Relatives Difficulty Interacting
____ 57. frnd/rel say look well when not
____ 58. frnd/rel withhold information
____ 59. frnd/rel avoid talk cancer
____ 60. frnd/rel do not visit enough
____ 61. frnd/rel do not call enough
____ 62. frnd/rel uncomfor visiting
____ 63. frnd/rel diff talk about cancer

### Anxiety in Medical Situations
____ 64. uncomfor see patients get treat
____ 65. nervous going to hospital
____ 66. nervous wait to see doctor
____ 67. nervous wait for test results
____ 68. nervous have diagnostic tests
____ 69. nervous get blood drawn

### Worry
____ 70. worry whether treatments work
____ 71. worry whether cancer progress
____ 72. worry not able to care for self
____ 73. worry how family will manage

### *Interaction with Children
____ 89. diff care for child/grandchild
____ 90. diff help children cope
____ 91. diff help children talk about illness

### *At Work Concerns
____ 92. diff talk boss about cancer
____ 93. diff talk people at work
____ 94. diff tell employer cannot do work
____ 95. diff ask time off for treatments
____ 96. worried about being fired
____ *SUM* ___ *#(1-4) 44, 41, 39, 36*
\# Potential Circle

## SEXUAL

### Sex Interest
____ 74. doesn't feel sex. attract
____ 75. thinks not sexually attractive to partner(s)
____ 76. not interested in having sex
____ 77. doesn't think partner(s) interested in sex

### *Sexual Dysfunction
____ 99. frequency of sex decreased
____ 100. diff become sexually aroused
____ 101. diff with erection (males)
____ 101. diff lubrication (females)
____ 102. diff reach orgasm
____ *SUM* ___ *#(1-4) 8, 4*
\# Potential Circle

* Items may not apply to all patients

## MISCELLANEOUS

### Compliance
____ 78. doesn't show for MD appoint
____ 79. doesn't show for treatments
____ 80. doesn't take medication
____ 81. doesn't follow MD's instruct
____ *SUM* ___ *#(1-4) 4 # Potential*

### Economic Barriers
____ 82. financial problems
____ 83. insurance problems
____ 97. diff find new job**
____ 98. employers no hire CA hist**
____ *SUM* ___ *#(1-4) 4, 2*
\# Potential Circle

### *Dating
____ 121. diff initiating dates
____ 122. diff meet dates
____ 123. afraid go places meet dates
____ 124. diff tell date about cancer
____ 125. afraid to initiate sex relation
____ *SUM* ___ *#(1-4) 5, 0*
\# Potential Circle

### *Chemotherapy-Related Problems
____ 126. nervous get chemo
____ 127. nauseated during/before chemo
____ 128. vomit during/before chemo
____ 129. sick when think about chemo
____ 130. nauseated after chemo
____ 131. vomit after chemo
____ 132. tired after chemo
____ 133. other side effects chemo
____ 134. lost hair/grow slow from chemo
____ *SUM* ___ *#(1-4) 9, 0*
\# Potential Circle

### *Radiation-Related Problems
____ 135. fatigued after rad
____ 136. nervous get rad
____ 137. nauseous/vomit after rad
____ *SUM* ___ *#(1-4) 3, 0*
\# Potential Circle

### *Ostomy
____ 138. problems ostomy care/maint.
____ *SUM* ___ *#(1-4) 1, 0*
\# Potential Circle

### *Prosthesis
____ 139. diff with prosthesis
____ *SUM* ___ *#(1-4) 1, 0*
\# Potential Circle

### Miscellaneous Items
____ 84. diff with transport
____ 85. gain too much weight
____ 86. diagnostic proced painful
____ 87. frequent diarrhea
____ 88. poor bladder control
____ *SUM* ___ *#(1-4) 5 # Potential*

## SUM ALL 8 MISCELLANEOUS SUMS ABOVE
____ *SUM* ___ *#(1-4)* ____ *# Potential*

## Global and Average Severity for CARES and 5 Subscales

| Scale | SUM | # Endor | # Poten | AVE Sever | GLOBAL |
|---|---|---|---|---|---|
| **Physical** | ____ | ____ | ____ | ____ | ____ |
| **Psychoso** | ____ | ____ | ____ | ____ | ____ |
| **Med Int** | ____ | ____ | ____ | ____ | ____ |
| **Marital** | ____ | ____ | ____ | ____ | ____ |
| **Sexual** | ____ | ____ | ____ | ____ | ____ |
| **Miscell** | ____ | ____ | ____ | ____ | ____ |
| **CARES** | ____ | ____ | ____ | ____ | ____ |
